# Quantitative cell imaging approaches to metastatic state profiling

**DOI:** 10.3389/fcell.2022.1048630

**Published:** 2022-10-25

**Authors:** Andres J. Nevarez, Nan Hao

**Affiliations:** Department of Molecular Biology, School of Biological Sciences, University of California, San Diego, San Diego, CA, United States

**Keywords:** metastasis, cellular morphology, quantitative imaging, light microscopy, machine learning, deep learning

## Abstract

Genetic heterogeneity of metastatic dissemination has proven challenging to identify exploitable markers of metastasis; this bottom-up approach has caused a stalemate between advances in metastasis and the late stage of the disease. Advancements in quantitative cellular imaging have allowed the detection of morphological phenotype changes specific to metastasis, the morphological changes connected to the underlying complex signaling pathways, and a robust readout of metastatic cell state. This review focuses on the recent machine and deep learning developments to gain detailed information about the metastatic cell state using light microscopy. We describe the latest studies using quantitative cell imaging approaches to identify cell appearance-based metastatic patterns. We discuss how quantitative cancer biologists can use these frameworks to work backward toward exploitable hidden drivers in the metastatic cascade and pioneering new Frontier drug discoveries specific for metastasis.

## Cannot grind and find them all: Genomics hits the metastatic wall

**FIGURE 1:**
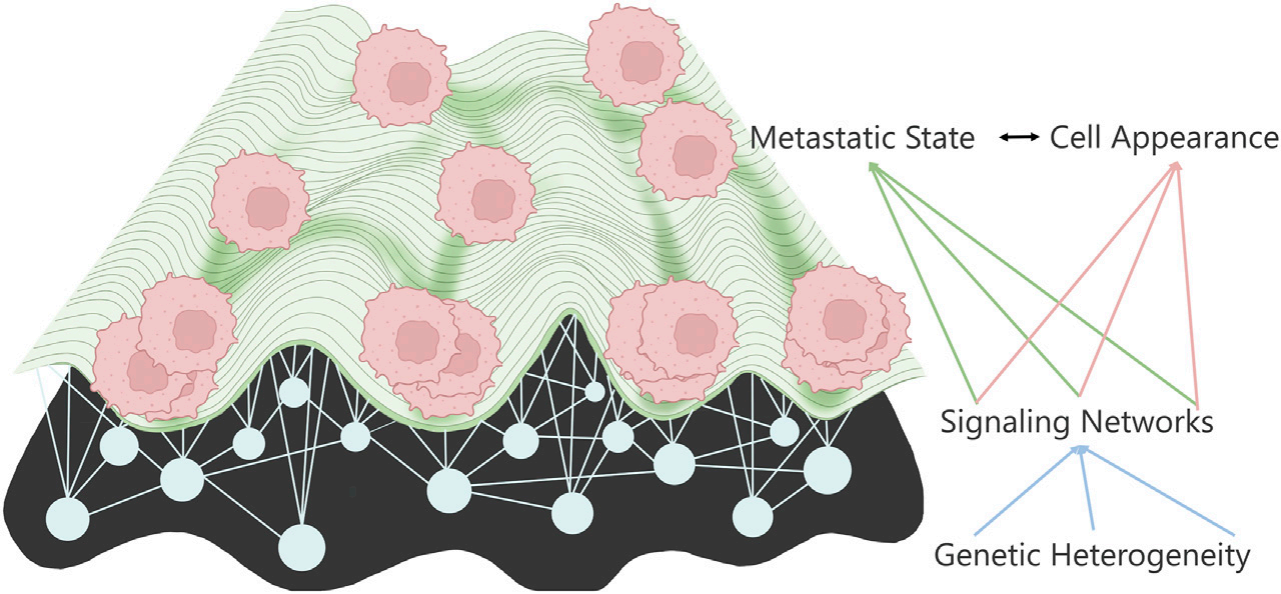
Metastatic landscape connects to cell appearance through the underlying signaling networks determined by the various mutations.

While we have made enormous progress regarding our understanding of cancer, it is still a leading cause of death worldwide. The cause of this high lethality is primarily due to the metastatic stage of the disease; metastasis occurs when cells from the primary tumor leave the local environment and colonize a distant organ (Bogenrieder and Herlyn, 2003; Gupta and Massagué, 2006; Chiang and Massagué, 2008; Hanahan and Weinberg, 2011; Reddy et al., 2012; Dillekås et al., 2016). Metastasis, and therefore therapy resistance, is the last Frontier of cancer treatment; it has been shown that metastasis and therapy-resistant cells share many common properties (Fares et al., 2020). However, less than 1% of the cells from the primary location can create tumors in distant organs. While metastatic cells may be rare events, they possess extraordinary abilities to survive an onslaught of insults that the cells must endure disseminating, colonizing, and thriving in a new microenvironment. Metastatic cells must possess cell properties that are entirely different from their primary, stationary counterparts. This way, metastatic cells adapt to evolutionary pressures (Schardt et al., 2005) by creating polyclonal populations, some of which survive each stressor (Cheung et al., 2016; Lo et al., 2020; Kok et al., 2021) to ultimately thrive in distinct niches (Gal et al., 2015; Piskounova et al., 2015; Tasdogan et al., 2020) from the primary tumor site. Surprisingly, the deluge of mutations from genomic studies has landed on stereotypical metastatic signaling states (Klein, 2009) reminiscent of the Waddington landscape (Waddington, 1959), although not converging on the metastatic target pathway. This has led the field to use phenotypic-driven interrogations of metastasis to work toward identifying the hidden drivers in the metastatic cell state (Figure 1).

## Appearances can be revealing

Subtle changes in metastatic cell states should manifest themselves in detectable phenotypic changes. This change is due to the morphological connection to the cytoskeleton changes necessary for invasion in the metastatic cascade. Initially, these changes were quantified through static shape morphometrics and connected to changes in metastatic potential. Cell appearance is a reliable monitor of cell signaling pathways (Bakal et al., 2007; Yin et al., 2013; Goodman and Carpenter, 2016; Gordonov et al., 2016; Pascual-Vargas et al., 2017; Sero and Bakal, 2017; Scheeder et al., 2018) due to the strong connection to the cytoskeleton (Moujaber and Stochaj, 2020), which is readout metastatic expression profiles (Nguyen et al., 2016), and a cell’s ability to invade (Minn et al., 2005). Recent studies have shown explicitly that cell appearance phenotypes have a solid connection to the metastatic phenotype (Cooper et al., 2015; Lyons et al., 2016; Wu et al., 2020). The use of morphological changes to identify invasive cancers stems from recognizing the epithelial to mesenchymal transition (EMT) (Li and Balazsi, 2018; Lu and Kang, 2019). Not only this, but pathologists often see gross morphological changes from primary to metastatic site biopsy samples, which have long been used for disease staging and grading (Lee et al., 2020). In this review, we highlight studies that not only pushed forward the morphological analysis of metastatic cells but offer an experimental and analytical platform, which are quantitative metastatic assays for probing the metastatic single-cell state.

## In the beginning, there were shapes: Two-dimensional cell morphology and machine learning to classify metastatic cells

Using two-dimensional (2D) cell culture has taught us the value of cell appearance and its relation to metastatic ability. Recent work has shown 2D cell shape to be a readout of metastatic cell state (Holenstein et al., 2019; Riehl et al., 2021). Lyons et al. showed a deep connection between shape and metastatic potential compared to paired non-metastatic parental cells (Lyons et al., 2016). They investigated the effect of three different surfaces with varying hydrophobicity; glass detergent washed and air-dried, glass acid-etched and air-dried, and siliconized ethanol-treated. Using four paired osteosarcomas, one with low metastatic potential and one with high metastatic potential from the same cell lineage, they showed that metastatic cells display different morphometric features using twenty-nine cellular and nuclear shape features. We must note that they focused on interpretable geometric shape features and not expansion shape features. They distilled these twenty-nine shape features into five morphological properties: projected cell size, cell roundness *versus* elongation, shape variability, nuclear size, and nuclear shape. They noted that high metastatic potential cells differ from their low potential counterparts in projected cell area and cell volume, which were experimentally validated. Examining only the nuclear shape features showed no low *versus* high metastatic potential trends. This highlights the pitfalls of using one morphometric as a classification tool, especially with prospects for clinical applications. However, they overcome this using machine learning feeding all shape features into a multilayer perceptron to classify cells. They showed their classifier had good accuracy against high and low cells from the same lineages across cell surfaces and aggregate classes such as high *versus* low metastatic potential. This early work, and others like it, laid the foundation for computer vision and machine learning techniques to investigate metastatic cells.

Wu and company have repeatedly continued the exploration of shape features in 2D. Building upon their work in pancreatic ductal adenocarcinoma (Wu et al., 2015), they investigated the heredity of morphological features in single-cell clones (SCC) of metastatic breast cancer cell lines. They found that each clone displayed a distinct morphology from which they investigated 14 SCC and their matched parental cell line. They quantified the cell morphology by extracting two hundred and fifteen cellular and nuclear morphometrics and then distilling them into seven distinct morphological profiles using unsupervised clustering methods. Within the clones, there was morphological heterogeneity, albeit at a much lower degree, compared to the parental line indicating there are heritable morphological traits. With these quantitative morphologies relating to cell appearance, they sought to see how the different morphologies affect metastatic potential *in vivo*. They implanted each of the seven morphological profiles into the mouse mammary fat pad and examined the metastatic potential of each morphology. They observed that depending on the morphology class, there were differences in metastatic potential and tumorigenesis compared to the parental line. They found a range of aggressiveness of the SCC: low tumorigenicity, tumorigenic, metastatic, and hypermetastatic. While they had several interesting findings regarding metastasis, tumor volume, and circulating tumor cells burden, they identified that cells with a high aspect ratio do not have higher metastatic potential, contradicting other findings in Lyons et al. However, SCCs with the same morphology displayed similar *in vivo* outcomes of tumorigenicity, circulating tumor cells, and metastatic potential; this highlights the connection between cellular appearance and metastasis. Stratifying the morphologies into high and low metastatic potential with corresponding gene expression profiles allowed Wu et al. to identify potential predictive metastasis genes for their cell line of choice. This highlights the immense impact that homogenous heritable morphological traits condense the heterogeneous genomic landscape to stereotypical cell morphologies.

## Functionalized coated cell surfaces further 2D morphological profiling of metastasis

Instead of using traditional plastic culture dishes or plain glass slides, Hasan et al. used 2D light microscopy of metastatic glioblastoma and astrocytes on functionalized glass coverslips (Hasan et al., 2018). They trained a supervised classifier with an accuracy of 82% to discriminate between non-cancerous astrocytes and metastatic glioblastoma cells taken from a patient biopsy. They previously developed a glass-coated coverslip with anti-EGFR aptamer, which showed a high affinity for cells that overexpress EGFR on the cell surface (Wan et al., 2010; Wan et al., 2013; Mahmood et al., 2015; Mansur et al., 2018). Building upon this in the current study, they laid the foundation to develop a framework to identify circulating tumor cells from blood samples in glioblastoma patients. The captured cells by the aptamer moved about over time while anchored to the coverslip to achieve short-term time-lapse imaging. They extracted multiple features such as area, perimeter, and center of mass of each cell for each time frame, each of which built different segmentation models such as aspect ratio, convexity, and best-fitted ellipse. They tracked the morphodynamic changes using the feature vectors of each segmentation model using the Hausdorff distance between time frames. This information was fed into three machine learning classifiers, Support Vector Machine (SVM), Naïve Bayes Classifier, and Random Forest Tree. They decided that the Naïve Bayes Classifier yielded the best classification results. They presented a unique and label-free approach that can be quickly implemented using standard instruments and low computational power in a clinical setting. The pipeline is essential for those that work with cells that are hard to transfect or for samples one may not want to perturb using fluorescent proteins.

Alizadeh et al. used fluorescent imaging of over a dozen cancer cell lines, with varying metastatic potential, on fibronectin-coated glass coverslips (Alizadeh et al., 2020). To discriminate between populations, they quantified the texture of the cell, the spreading of the cell, and irregular cell shape, all of which were fed into an SVM or shallow layered Perceptron. They found that cell and nuclei geometric shape features, interpreted as cellular spread size, elongation, and boundary irregularity, reliably represented a cell over experimental replicates and cell types. However, label-free features such as texture are even more reliable representations of cell states. Of note in this study was the comparison of high (MDA-MB-231), low metastatic (MCF7), and normal breast cells (MCF10A) and a range of linearly progressed matched osteosarcoma cancer cell lines. Using the extracted morphological information in the reduced principal component (PCA) space, they showed no linear progression from normal to low then high metastatic morphological space; instead, there is some overlap in each morphometric category. For instance, in cell hull geometry and waviness (Fourier transform) of the normal breast cells lie in the space between low and high metastatic potential breast cancer cell lines. However, the low metastatic potential lies between normal and high metastatic features for grayscale morphological features. For the osteosarcoma cell lines, grayscale and cell geometry features showed high separation between the normal and osteosarcoma cancer lines.

**FIGURE 2:**
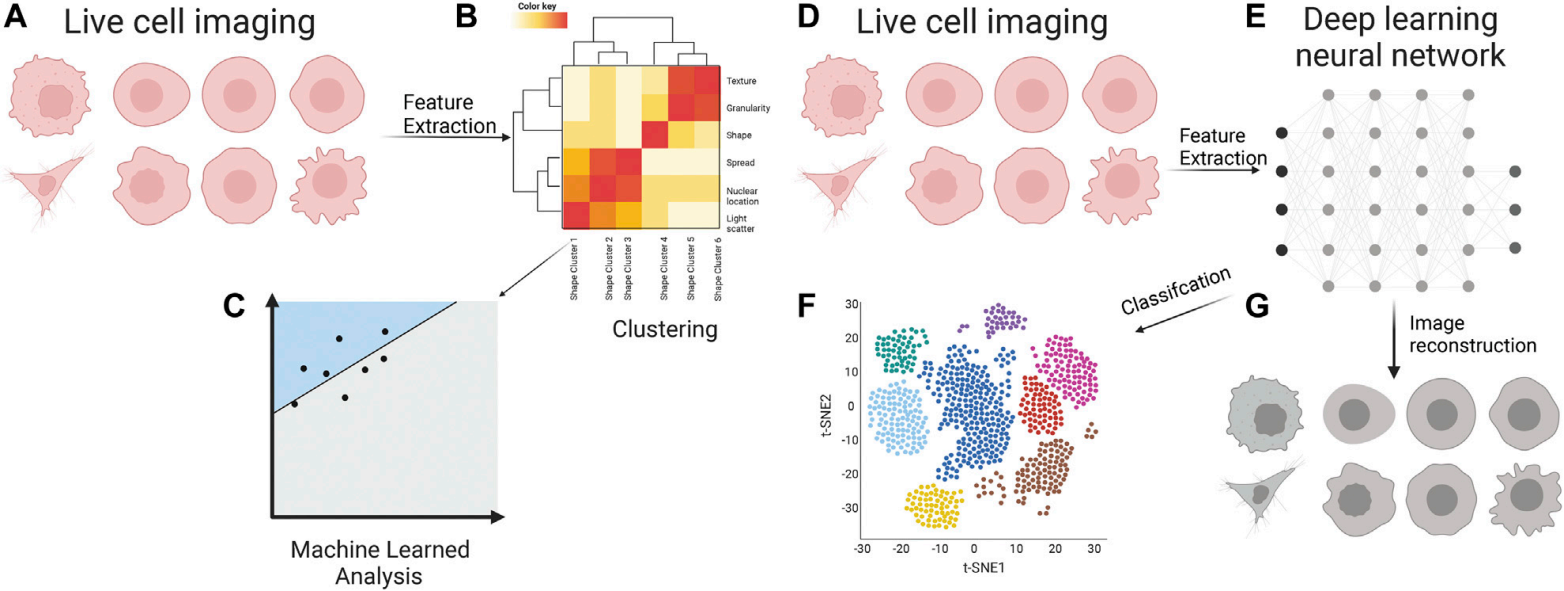
Cell appearance from live cell imaging **(A)** is quantified through supervised feature extraction, consisting of dozens of features that describe the cell appearance in many ways. Often these are clustered **(B)** into classes which feed into a machine-learned model for classification **(C)**. Live cell images **(D)** are inputs for various deep learning neural networks **(E)** for unsupervised feature extraction. The features are used for the classification of metastatic cells **(F)**. These features are often a black box of unknown representation of cell appearance; however, there are methods to decipher the critical cellular properties through image reconstruction **(G)**.

In contrast, waviness and hull geometry highly overlap all cell lines. This led them to test the morphological features at the single-cell level using an SVM or Perceptron. They found that morphometric-based features are more feature-rich for classification. Based on this, they can discriminate between the high and low metastatic cells of osteosarcoma and breast cancer cell lines. This led them to conclude that there may be stereotypical morphological transformations in the metastatic process. However, based on this study alone, it cannot be concluded how many stereotypical categories there may be for metastatic cells. This leaves an opportunity for long-term morphometric analysis of the development of normal to metastatic cells over many cancer types (Figure 2).

## 2.5D imaging and interpretable deep learning

Zaritsky et al. used label-free imaging and interpretable deep learning to identify cellular properties that discriminated between high and low metastatic potential patient-derived xenografted (PDX) melanoma cells (Zaritsky et al., 2021). While not strictly 2D, they imaged the PDXs atop a thick collagen matrix to negate the physical forces of a plastic/glass and the morphological homogeneity plastic culture dishes impose. They imaged the metastatic cells over multiple time durations to gather morphological dynamics. They noticed that metastatic cells were not particularly migratory and were defined mainly by rounded shape, with surface membrane blebbing, regardless of metastatic potential; this is consistent with other studies’ patient biopsies atop collage (Sadok et al., 2015). They hypothesized that neither shape nor migratory ability would yield discriminative power between high and low metastatic cells. Instead, the discriminative power may come from visually unstructured information in the images; to this end, they developed interpretable deep learned models. They developed an autoencoder, which comprises a deep convolutional neural network (CNN) to encode the unstructured latent cell information contained in the images. From this, they identified a latent cell descriptor that contains a compressed version of single-cell image information.

Using this latent cell descriptor, they could discriminate between a panel of metastatic melanoma cell lines against fetal foreskin melanocytes. Single-cell clones were created within the metastatic cell lines, and the latent descriptor could discern the parental line from the clonal line. Furthermore, they could successfully discriminate between the cell line and PDX metastatic melanoma panels. Incongruent with the other studies mentioned in this review, cell shape performed worse than the latent cell descriptor, and temporal information did not increase the latent descriptor’s discriminative power. They could discriminate between high and low metastatic melanoma PDXs using the latent cell descriptor and linear discriminate analysis machine learning classifier. While exploring existing deep learning models on a unique physiologically relevant 2D system, the authors investigated the latent descriptor encoded in cell properties. They identified that pseudopodial extensions and interior light scattering properties of the cell discriminate between high and low metastatic melanoma. It is important to note that many studies focused on using machine learning and deep learning methods to identify differences in morphologies of metastatic cells; most studies have not used interpretable methods. This is severely lacking in most investigations, yet it is critical to work backward from phenotype to actionable signaling targets.

## Integrating 3D cell morphology and dynamic information for metastatic profiling

While static imaging in 2D has profoundly impacted the field, it was always apparent that those studies were missing the mechanical forces that affect the cytoskeleton and, thus, the signaling pathways to which it connects. Furthermore, stereotyped cell behaviors can be identified from cell actions imaged over time (Tweedy et al., 2019). Here we focus on studies that have exploited three-dimensional (3D) and time-lapsed imaging of dynamic morphological changes.

Elbez et al. developed a unique approach to image single-cell dynamic morphological phenotypes using machine learning and magneto-rotation, simulating circulating tumor cell morphologies in 3D. (Elbez et al., 2021). They developed magnetic nanoparticles that are endocytosed into the cell, which activated green-fluorescent protein (GFP). They used an external oscillating magnetic field to suspend and rotate cells with the nanoparticles in a microfluidic device so they could image 3D morphological deformation. Using the supervised Adaboost machine learning method of single cells (identified and segmented using GFP), they were able to identify metastatic cells (MDA-MB-231) that had undergone the EMT, cells that were not metastatic (MCF-7) with an f1 score of 0.965. Next, they used the prostate cancer cell line PC-3 as a control, then forced PC-3 cells to undergo EMT and become HR-14 cells to test if their classifier could identify the same cell lineage. Still, they could differentiate between the two states of the same cell lineage with different cell states. They used the unsupervised K-means clustering method (with a strict homogeneity score of 0.95) to identify seven distinct morphological phenotypes within the populations. They followed up on this and determined they could differentiate between functional phenotypes of high and low migratory and invasive cells using the MDA-MB-231 cell line and a Boyden chamber. Unfortunately, they do not identify the morphological phenotypes that either machine-learned method used to discriminate between the populations. However, this can be remedied using interpretable learning methods.

Recently it was observed that metastatic breast cancer cell lines embedded in collagen exhibited morphological phenotype transitions that allowed them to efficiently traverse non-uniform matrixes that mimic the ECM (Eddy et al., 2021). They developed machine learning models to quantify cell shape dynamics in 3D for up to 24 h. Eddy et al. quantified cell appearance using twenty-one shape geometric features, including cell size, backbone curvature, surface topography, and deviation from circle shape. They found the geometric space of the cells sample is similar to random walk; however, they found the morphology dynamics are subdiffusive while having superdiffusive properties in actual 3D space. Interestingly, they found the same cells exhibited increased morphodynamics sampling of geometric space on 2D surfaces than 3D embedded in ECM; this again highlights meaningful differences between 3D and 2D morphological analysis. Using manually labeled cells, they trained an SVM to classify them into four distinct cell morphological phenotypes, with an accuracy of 88%. They classified the four morphological phenotypes: actin-enriched leading edge, small blebbing, filopodial, and lamellipodial. They focused on these four morphological phenotypes due to their tight connection to molecular profiles: actin-enriched leading edge has elevated actin protrusions; small blebbing has high cortical stress, which drives the blebbing; filopodial and lamellipodial phenotypes have strong ECM adhesions with polarized bodies, where the filopodia distinguish themselves with F-actin bundles running across the cell body, while the lamellipodia distinguish itself through cellular fan shapes. They perturbed the signaling networks attached to these phenotypes and changed the ECM homogeneity. Focusing on disrupting the RHO/Rock signaling pathway, they discovered that perturbations did not force cells to favor one phenotype over another. Instead, it altered the morphodynamics of phenotypes. Decreasing RHO expression led to amoeboid-to-mesenchymal transitions through the actin-enrich and lamellipodial phenotypes.

In contrast, activation of RHO led to increased morphodynamics overall, which enriched the blebbing morphological phenotype. Not only does this shed light on the morphological plasticity to traverse heterogeneous ECM through phenotype switching, but this study also highlights the interpretable use of machine learning critical for furthering metastatic research. Using morphology, the quantified cell appearance differences have been connected to enhanced cell cycle progression, especially important for micro-metastases (Mohan et al., 2019; Molinie et al., 2019). Multiple studies have followed up on their metastatic cell appearance metrics *in vivo* using a mouse model. Indeed, these cell appearance changes identified using A.I. have translated to *in vivo* metastatic potential. While it may seem evident that anti-cytoskeletal therapies will inhibit metastasis (Fife et al., 2014; Gandalovičová et al., 2017; Aseervatham, 2020), these drugs often are non-specific to metastatic tumors, are highly cytotoxic and cardiotoxic (Stehn et al., 2013), and often fail to suppress metastasis. It is believed this is due to cytoskeletal plasticity, highlighted in Eddy et al.

Furthering the interrogation between 3D morphology and metastatic cell state, Driscoll et al. developed u-shape3D, which identifies morphological motifs of cells migrating through microenvironments using high-resolution 3D light sheet microscopy (Driscoll et al., 2019). U-shape3D was a launching point for Segal et al. to investigate the morphological motif changes that metastatic Ewing Sarcoma cells undergo while migrating through various microenvironments in the Zebrafish model system (Segal et al., 2022). They found that the two Ewing Sarcoma cell lines have single-cell morphological distribution changes as the cell migrates through different parts of the Zebrafish. To understand the meaning of these morphotype changes, they mapped the single-cell morphotypes to clusters in the Principal Component space and qualitatively described these clusters for interpretability. They found that the TC32 cell line shared morphotypes in the perivitelline space (PVS) and caudal hemopoietic tissue (CHT). In contrast, cells in the hindbrain ventricle (HBT) exhibited more protrusions and larger cell shapes. While TC71 distributions in the PVS and HBV shifted to a rounded morphotype compared to cells in the CHT. When investigating the effect of changes in EF1 expression, the gene fusion which characterizes Ewing Sarcoma, they found that loss of EF1 led to site-specific morphotype changes within the Zebrafish. This study highlights that the expression landscape will affect the cells’ morphological plasticity in response to various microenvironments *in vivo*.

## Future prospective and concluding remarks

The diagnostic applications derived from these approaches will allow clinicians to identify metastatic cells in biopsy samples. While not used previously in identifying metastatic cells, we want to highlight Imaging Flow Cytometry (IFC). The pairing of deep learning and machine learning with different imaging technologies, such as IFC, can interrogate the metastatic stage in primary samples as part of the pathology pipeline. Given that these samples can be fragile and genetic engineering can perturb the cell state, IFC can probe the cell using cell light scatter and brightfield images, both of which require no labels and have been used for cancer classification with deep and machine learning.

We have highlighted multiple studies investigating cell appearance in varying spatiotemporal imaging modalities as a readout of metastatic potential to accelerate the discovery of metastatic-specific features. Rather than focusing on a specific gene, these works have levied the rich information found in images of metastatic cancer cells, overcoming the limits of using the genetic code for metastatic profiling.
